# Comparison of 1-year outcomes after Ahmed glaucoma valve implantation with and without Ologen adjuvant

**DOI:** 10.1186/s12886-018-0709-2

**Published:** 2018-02-14

**Authors:** Tai Jun Kim, Sohyun Kang, Jin Wook Jeoung, Young Kook Kim, Ki Ho Park

**Affiliations:** 1 Department of Ophthalmology, Seoul National University Hospital, Seoul National University College of Medicine, 101 Daehak-ro Jongno-gu, Seoul, 03080 Republic of Korea; 2 University of Rochester Medical Center, Rochester, NY USA

**Keywords:** Ologen, Early hypertensive phase, Ahmed glaucoma valve, Glaucoma

## Abstract

**Background:**

Many studies have investigated the clinical benefits of Ologen for trabeculectomy. However, its benefits for Ahmed glaucoma valve (AGV) implantation have not been investigated as extensively. The aim of this study was to compare the 1-year outcomes of AGV implantation with and without Ologen adjuvant for the treatment of refractory glaucoma.

**Methods:**

This retrospective study included a total of 20 eyes of 20 glaucoma patients, who were followed for at least 1-year after undergoing AGV implantation. In 12 eyes of 12 patients, conventional AGV (CAGV) surgery was performed, while in 8 eyes of 8 patients, Ologen-augmented AGV (OAGV) implantation was performed. The outcomes were evaluated according to intraocular pressure (IOP) and the number of IOP-lowering medications. Complete success was defined as IOP ≤ 21 mmHg without medications throughout the 1-year follow-up period, and qualified success was defined as IOP ≤ 21 mmHg with or without medications throughout the 1-year follow-up period.

**Results:**

The rate of complete success was significantly higher in the OAGV group (50.0%) than in the CAGV group (8.3%) (*p* = 0.035). There were no significant differences between the two groups in terms of qualified success or incidence of the early hypertensive phase. The IOP changes were similar between the groups within 1-year postoperatively, though the number of IOP-lowering medications was significantly lower in the OAGV group during the early hypertensive phase (*p* = 0.031, 0.031, and 0.025 at postoperative months 1, 2, and 3, respectively). When subjects were divided into groups according to the occurrence of the early hypertensive phase, the group with early hypertensive phase was more likely to use IOP-lowering medications at postoperative 6 months and 1 year (*p* = 0.002 and 0.005, respectively).

**Conclusions:**

OAGV surgery shows encouraging results for patients with refractory glaucoma, specifically with respect to the achievement of complete success and the reduction of the number of IOP-lowering medications during the early hypertensive phase. Furthermore, our results suggest that occurrence of the early hypertensive phase is predictive of which patients will require IOP-lowering medications at postoperative 6 months and 1 year.

## Background

Trabeculectomy being the most commonly performed surgical procedure for intraocular pressure (IOP) lowering, the use of glaucoma drainage devices (GDD) has historically been limited to refractory glaucoma – which is to say, high-risk cases such as secondary or neovascular glaucoma that are unresponsive either to medical therapy or conventional surgical intervention [1, 2]. However, since the publication of the “Tube Versus Trabeculectomy” paper, which reported a landmark study showing a higher long-term success rate and a lower complication rate for GDD compared with trabeculectomy with Mitomycin C (MMC), the role of GDD has been expanding to a wider range of patients [3–5].

Histological studies have shown the efficacy of trabeculectomy to be limited by the proliferation of subconjunctival fibroblasts and subsequent scarring of the filtering bleb, which necessitates the use of adjunctive antimetabolites such as 5-fluorouracil and MMC for prevention of scar formation [6–8]. However, due to the increased risk of complications such as bleb leakage, hypotony, and infection, a biodegradable collagen matrix implant known as Ologen (Aeon Astron Europe B.V., Leiden, The Netherlands) has grown in popularity as an alternative method for enhanced trabeculectomy success [9]. Compared with MMC, Ologen has shown comparable results for trabeculectomy; indeed, it represents a potentially new way of managing IOP and its complications in glaucoma surgeries [10–13].

Whereas many studies have investigated the clinical benefits of Ologen for trabeculectomy, its benefits for GDD have not been investigated as extensively. Among GDD, the Ahmed glaucoma valve (AGV) is widely used due to its effectiveness in reducing the occurrence of postoperative hypotony and the related complications [14–17]. Despite its efficacy however, AGV has been criticized for its induced IOP increase during the early hypertensive phase, a postoperative period of usually 1–2 months which resolves within 6 months [18, 19]. Recently, Rho et al. [20] investigated the effect of Ologen on AGV surgeries with a 6-month follow-up period, but the type of Ologen that had been used had a different rectangular shape and dimensions (10 mm × 10 mm × 2 mm). Song et al. [21] also investigated clinical and biological aspects of Ologen on AGV surgeries with at least 6 month follow-up period, however, not only group setting (cylindrical Ologen group vs rectangular Ologen group vs control group) but also the number or shape of Ologen (two cylindrical Ologens vs one rectangular Ologen) that had been used were different. (In contrast, we used only one cylindrical Ologen that more closely fits the subconjunctival space and does not require suturing to the AGV to maximize the plasticity of the collagen matrix.)

Given Ologen’s potential for efficacy and safety in trabeculectomy, our study aimed to explore the effect of a cylindrical Ologen in the management of the early hypertensive phase in AGV implantation.

## Methods

This study followed the tenets of the Declaration of Helsinki, and the research protocol was approved by the Institutional Review Board of Seoul National University Hospital. This retrospective study included 8 consecutive cases of Ologen-augmented AGV (OAGV) surgery in 8 eyes of 8 subjects and 12 matched control cases of conventional AGV (CAGV) surgery in 12 eyes of 12 subjects, all performed to treat refractory glaucoma at Seoul National University Hospital between March 2011 and October 2016.

Refractory glaucoma was defined as insufficient IOP control (IOP > 21 mmHg) or progressive visual field deterioration despite maximally tolerated anti-glaucoma medical therapy. Patients younger than 18 or with previous history of AGV surgery (but not other types of glaucoma surgery such as trabeculectomy) were excluded, and all of the patients were followed for at least 1-year postoperatively.

**Fig. 1:**
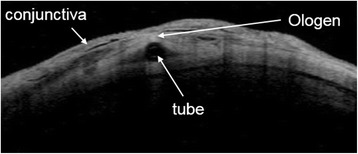
AS-OCT image of bleb over Ologen on postoperative day 1. Healthy bleb morphology and aqueous filtering under the conjunctiva are shown

Preoperatively, all of the eligible patients received complete ophthalmic examinations, including slit lamp examination, IOP measurement with Goldmann’s applanation tonometry, dilated fundus examination, and gonioscopy. Postoperative visits were scheduled for day 1, months 1, 2, 3, 6 and year 1. At each visit, IOP, biomicroscopic findings, number of IOP-lowering medications, and postoperative complications were assessed. Also, anterior segment-optical coherence tomography (AS-OCT) (Carl Zeiss, Jena, Germany) was utilized to evaluate the bleb morphology in one subject from the OAGV group (Fig. 1).

Outcomes were primarily determined by IOP and the number of IOP-lowering medications. Complete success was defined as IOP ≤ 21 mmHg without any IOP-lowering medication throughout the 1-year follow-up period. Qualified success was defined as IOP ≤ 21 mmHg with or without IOP-lowering medications throughout the 1-year follow-up period. Early hypertensive phase was defined as IOP greater than 21 mmHg not caused by tube obstruction, retraction, or valve malfunction within postoperative 3 months (with or without medications) after reduction to less than 22 mmHg during the first postoperative week.

### Surgical techniques

All of the surgeries were performed by a single experienced surgeon (Park KH). A fornix-based conjunctival incision at the limbus and along with two radial incisions was made in the superotemporal quadrant with blunt Westcott scissors. Blunt dissection was performed to clear a space on the sclera in the quadrant. A rectangular-shape limbus-based scleral flap was made to cover the tube of the AGV. The AGV was primed by flushing balanced salt solution through the tube to confirm patency. Only in the OAGV group, a round 12 mm (D) × 1 mm (H) Ologen (model 862,051, Aeon Astron Europe B.V., Leiden, The Netherlands) was positioned on the plate of the AGV without any fixation suturing and inserted into the sub-Tenon’s space over the sclera. The plate was fixed to the sclera with two 8–0 nylon sutures (Ethilon, Ethicon, Inc., Somerville, NJ, USA). The tube was placed under the scleral flap, and its tip was obliquely cut bevel-up to a length at which the tube could properly extend into the anterior chamber. Afterwards, 8–0 polyglactin suturing (Vicryl, Ethicon, Inc., Somerville, NJ, USA) was used to close the scleral flap as well as the conjunctiva after it was approximated to the limbal area. Upon completion of the surgery, dexamethasone disodium phosphate (Dexamethasone, Daewon, Seoul, Korea) was injected subconjunctivally and the following postoperative medications were prescribed in all cases: 0.5% levofloxacin (Cravit, Santen, Osaka, Japan) and 0.5% loteprednol etabonate (Lotemax, Bausch & Lomb Pharmaceuticals, Inc., Tampa, FL, USA) 4 times daily for 4 weeks, and 1% atropine sulfate (1% Isopto atropine, Alcon, Fort Worth, TX, USA) 2 times daily for 1 week.

### Statistical analysis

Data were statistically described in terms of mean ± SD, median frequencies (number of cases), and percentages, when appropriate. The Mann–Whitney U-test was used for comparison of the numerical variables between the study groups, while the χ^2^-test (or Fisher’s exact test) was utilized for the categorical data. All *p*-values less than 0.05 were considered statistically significant. The data were digitized and analyzed using Microsoft Excel 2013 (Microsoft Corporation, Redmond, WA, USA), and all of the statistical calculations were performed using SPSS (SPSS 18.0, Chicago, IL, USA).

## Results

As Table 1’s demographic data indicate, all of the baseline characteristics were similar between the two groups. Subjects with a history of AGV surgery were excluded, though 50% of those in each group (4 in OAGV group, 6 in CAGV group) had a history of either trabeculectomy or trabeculotomy. In the present study, none of the eyes developed any vision-threatening complications postoperatively.

**Table 1:** Demographic characteristics of subjects

	OAGV group (*n* = 8)	CAGV group (*n* = 12)	*p*-value
Age, years (mean ± SD)	60.9 ± 19.6	63.1 ± 14.1	0.910^a^
Female, patients (%)	4 (50.0%)	6 (50.0%)	0.350^b^
Diabetes, patients (%)	1 (12.5%)	4 (33.3%)	0.255^b^
Hypertension, patients (%)	2 (25.0%)	2 (16.7%)	0.381^b^
Diagnosis, eyes (%)			0.951^b^
Neovascular glaucoma	1 (12.5%)	2 (16.7%)	
Uveitic glaucoma	1 (12.5%)	1 (8.3%)	
Pseudoexfoliation glaucoma	1 (12.5%)	3 (25.0%)	
Primary open-angle glaucoma	2 (25.0%)	4 (33.4%)	
Congenital glaucoma	2 (25.0%)	1 (8.3%)	
Primary angle-closure glaucoma	1 (12.5%)	1 (8.3%)	
Previous glaucoma surgery, eyes (%)	4 (50.0%)	6 (50.0%)	1.000^b^
Preoperative IOP (mean ± SD)	33.4 ± 6.3	30.6 ± 6.8	0.238^a^
Number of preoperative IOP-lowering medications (mean ± SD)	3.6 ± 1.2	4.0 ± 1.2	0.473^a^

^a^Mann-Whitney U-test; ^b^χ^2^-test; *OAGV* Ologen-augmented Ahmed glaucoma valve implantation, *CAGV* conventional Ahmed glaucoma valve implantation, *SD* standard deviation, *IOP* intraocular pressure

The success rates and occurrence of early hypertensive phase in the study groups are reported in Table 2. The complete success rate was significantly higher in the OAGV group (50.0%) than in the CAGV group (8.3%) (*p* = 0.035). The incidences of the early hypertensive phase, 12.5 and 33.3% in the OAGV and CAGV groups respectively, as well as the rates for qualified success, 75.0 and 50.0% respectively, did not show any statistical significance.

**Table 2:** Success rate and early hypertensive phase frequency in both groups

	OAGV group	CAGV group	*p*-value
Complete success	4 (50.0%)	1 (8.3%)	0.035^a^
Qualified success	6 (75.0%)	6 (50.0%)	0.264^a^
Early hypertensive phase	1 (12.5%)	4 (33.3%)	0.375^b^

^a^χ^2^-test; ^b^Fisher’s exact test; *OAGV* Ologen-augmented Ahmed glaucoma valve implantation, *CAGV* conventional Ahmed glaucoma valve implantation

**Fig. 2:**
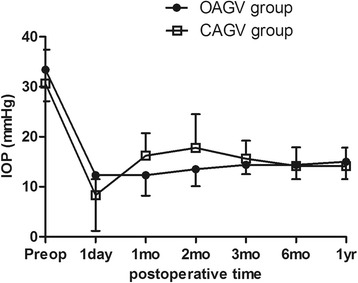
Mean IOP follow-up during postoperative 1-year in OAGV and CAGV groups. At every point, the inter-group difference failed to meet the threshold for statistical significance

Figure 2 plots the collective IOP measurements from each postoperative visit (day 1, months 1, 2, 3, 6, and year 1). Mean IOP in CAGV group is slightly numerically higher, though the inter-group difference was not statistically significant throughout the follow-up period (*p* = 0.571, 0.069, 0.115, 0.521, 0.910, and 1.000 at postoperative day 1, months 1, 2, 3, 6, and year 1, respectively). During the early hypertensive phase, however, the number of IOP-lowering medications used was significantly decreased in the OAGV group (*p* = 0.031, 0.031, and 0.025 at postoperative months 1, 2, and 3, respectively; Table 3). These results can be explained by the fact that the IOP difference between the two groups during the early hypertensive phase, though not statistically significant, was large enough to influence the management of the early hypertensive phase with IOP-lowering medications in different patients. When we divided subjects into two groups based on occurrence of the early hypertensive phase, the group that underwent early hypertensive phase was more likely to use IOP-lowering medications at postoperative 6 months and 1 year (*p* = 0.002 and 0.005, respectively; Table 4).

**Table 3:** Numbers of IOP-lowering medications at follow-up in both groups

	OAGV group	CAGV group	*p*-value
Day 1	0.5 ± 1.3	0	0.678^a^
Month 1	0.5 ± 1.0	2.1 ± 1.4	0.031^a^
Month 2	0.8 ± 1.1	2.3 ± 1.4	0.031^a^
Month 3	0.9 ± 1.4	2.3 ± 1.3	0.025^a^
Month 6	1.1 ± 1.7	2.5 ± 1.3	0.082^a^
Year 1	1.3 ± 1.6	2.3 ± 1.3	0.173^a^

^a^Mann-Whitney U-test; *IOP* intraocular pressure, *OAGV* Ologen-augmented Ahmed glaucoma valve implantation, *CAGV* conventional Ahmed glaucoma valve implantation

**Table 4:** Comparison of numbers of IOP-lowering medications according to occurrence of early hypertensive phase

	EHP (−) group (*n* = 15)	EHP (+) group (*n* = 5)	*p*-value
Month 1	0.8 ± 1.1	3.4 ± 0.5	0.001^a^
Month 2	1.0 ± 1.1	3.6 ± 0.5	0.001^a^
Month 3	1.2 ± 1.2	3.8 ± 0.4	0.000^a^
Month 6	1.3 ± 1.3	3.8 ± 0.4	0.002^a^
Year 1	1.3 ± 1.3	3.8 ± 0.4	0.005^a^

^a^Mann-Whitney U-test; *IOP* intraocular pressure, *EHP* early hypertensive phase

## Discussion

Our study suggests that compared with the CAGV procedure, OAGV surgery not only results in a significantly higher rate of complete success but also reduces the number of IOP-lowering medications that need to be used during the early hypertensive phase. Although our data showed results similar to those of recently published paper on the efficacy of Ologen in AGV surgeries [20], the type of Ologen we used in our surgeries was markedly different in terms of shape and dimensions.

In addition, Song et al. [21] demonstrated that the different biological properties of Ologen could affect the clinical outcome of AGV surgeries. Due to its different nature of the study design, we cannot establish a relationship between surgical success rate and the type of Ologen. According to the manufacturer, Ologen’s porous structure forces conjunctival fibroblasts and myofibroblasts to grow into the pores and secrete connective tissue in the form of a loose matrix, thereby reducing scar formation and wound contraction. We hypothesized that Ologen can actually serve as a mechanical scaffold for 90–180 days, inducing a potential subconjunctival volume and thus guaranteeing sufficient bleb space during the early hypertensive phase. Ologen’s role in forming a highly functional filtering bleb, therefore, can be the main cause of the higher complete success rate observed in the present OAGV group, and a secondary consequence could be that group’s decreased number of IOP-lowering medications used during the early hypertensive phase.

Although the difference in the rate of complete success between the two groups was statistically significant, there were no other significant differences in terms of other variables such as the rate of qualified success, the occurrence of the early hypertensive phase, or IOP changes during the 1-year postoperative follow-up period. The early hypertensive phase after AGV-FP7 implantation is known to occur in about 40–80% of cases [2, 9, 13, 15, 16]. It has been shown to be associated with a higher final IOP and, accordingly, to require a significant number of IOP-lowering medications for long-term IOP control [18]. Although the incidence of the early hypertensive phase was lower in the OAGV group than in the CAGV group, the difference was not significant; the incidence in the CAGV group was 33.3%, lower than the previously reported rate. As a result, the final postoperative IOP, which usually follows the early hypertensive phase, likewise did not show a significant difference between the two groups.

After postoperative 6 months, no significant difference in the number of IOP-lowering medications was observed between the two groups, which might be due to the fact that Ologen biodegrades within 6 months. If there is a way to slow down the degradation process of Ologen or to improve the delivery of mediators to enhance the modulation of wound healing, we speculate that such changes could not only reduce postoperative complications but also improve overall outcome of AGV surgery. Previous literatures have shown AGV surgery associated complications; hypotony (0%–3%), tube exposure (2%–14.3%), corneal complications (9%–27%), and endophthalmitis (0.8%–6.3%) [19]. However, in our small number of cases with 1 year follow-up, we did not experience any of those vision-threatening complications. Late failing AGV filtering bleb has been successfully managed by 5-fluorouracil needling revision [22]. In our case series, we did not perform the bleb needling.

There are several limitations to our study. First, because of our relatively small sample size and the retrospective nature of the study design, the results might include bias and, therefore, might not be completely representative of the general population. Second, we did not use the AS-OCT to evaluate the filtering bleb in all of subjects. If we had, in fact, obtained cross-sectional images of the surgical site in every subject, the bleb and conjunctival morphology could further elucidate Ologen’s role in AGV surgery. Third, randomization was not performed when dividing the subjects into OAGV and CAGV groups, because the purpose of the study was to compare the result of consecutively performed OAGV surgery and that of existing CAGV groups. Therefore, a prospective randomized controlled study will be needed in the future.

## Conclusions

OAGV surgery showed, relative to the CAGV procedure, a significantly higher complete success rate and an effective reduction of the number of IOP-lowering medications within postoperative 3 months. Moreover, our results imply that occurrence of the early hypertensive phase is predictive of which patients will require IOP-lowering medications at postoperative 6 months and 1 year. However, due to the relatively small sample size of the current study, further clinical trials with larger patient cohorts are required in order to reveal the effects of the Ologen adjuvant in AGV surgery.

## Abbreviations

AGV: Ahmed glaucoma valve
AS-OCT: Anterior segment-optical coherence tomography
CAGV: Conventional AGV
GDD: Glaucoma drainage devices
IOP: Intraocular pressure
MMC: Mitomycin C
OAGV: Ologen-augmented AGV
